# Cytokine profile characterization of naïve patients with psoriasis and psoriatic arthritis: implications for a pathogenic disease continuum

**DOI:** 10.3389/fimmu.2023.1229516

**Published:** 2023-07-13

**Authors:** Piero Ruscitti, Maria Esposito, Ilenia Di Cola, Cristina Pellegrini, Andrea De Berardinis, Mirco Mastrangelo, Camilla Gianneramo, Antonio Barile, Maria Concetta Fargnoli, Paola Cipriani

**Affiliations:** Department of Biotechnological and Applied Clinical Sciences, University of L’Aquila, L’Aquila, Italy

**Keywords:** psoriatic disease, psoriasis, psoriatic arthritis, mechanistic biomarkers, biomarkers

## Abstract

**Background:**

The idea of psoriatic disease continuum has been progressively prompted based on the advances of the knowledge about the pathogenic steps underpinning the occurrence of psoriasis (PSO) and psoriatic arthritis (PSA). To evaluate biomolecules (inflammatory cytokines, inflammatory chemokines, cell adhesion and cellular mediators) in naïve patients with PSO, PSA with PSO, and PSA sine PSO. To stratify the results considering the presence of psoriatic nail involvement, extensive skin disease and obesity evaluating all involved patients.

**Methods:**

By multiplex technology, 20 serum biomolecules were assessed with the inclusion of pro-inflammatory cytokines (GM-CSF, IFN-γ, IL-1α, IL-1β, IL-6, IL-8, IL-12p70, IL-17A, IL-23, TNF), anti-inflammatory cytokines (IFN-α, IL-4, IL-10, IL-13), inflammatory chemokines (IP-10, MCP-1, MIP-1α, MIP-1β), cell adhesion and cellular mediators (ICAM-1, E-selectin, P-selectin). The assessment of possible statistical differences between the means of the three groups was performed by One-Way ANOVA. In addition, by non-parametric T-tests, we stratified the results according to selected clinical characteristics (psoriatic nail involvement, PASI ≥ 10, BMI ≥ 30).

**Results:**

In 80 assessed naïve patients, patients with PSO showed significant increases of E-selectin (p=0.021) and IL-8 (0.041) than other groups. In patients with PSA with PSO, significant higher levels of ICAM-1 were observed (p=0.009) than other groups. We did not observe further differences comparing pro-inflammatory and anti-inflammatory cytokines, inflammatory chemokines, and cell adhesion and cellular mediators in patients with PSO, PSA with PSO, and PSA sine PSO. Patients with psoriatic onychopathy showed significant increased levels of ICAM-1 (p=0.010) and IP-10 (0.030) than others. In patients with PASI ≥ 10, significantly enhanced values of IL-8 (p=0.004), TNF (p=0.013), E-selectin (p=0.004), MIP-1α (p=0.003), and MIP-1β (p=0.039). In patients with BMI ≥ 30, significantly higher levels of E-selectin were pointed out (p=0.035) than others.

**Conclusion:**

Our findings may suggest that a similar cytokine profile may characterize naïve patients with PSO, PSA with PSO, and PSA sine PSO, reinforcing the concept of psoriatic disease continuum. However, some differences may be also shown, underlying possible pathogenic differences and leading to the clinical heterogeneity of these patients.

## Introduction

The idea of psoriatic disease continuum has been progressively prompted based on the advances of the knowledge about the pathogenic steps underpinning the occurrence of psoriasis (PSO) and psoriatic arthritis (PSA) (1). PSO is an inflammatory skin disease characterized by erythematous and scaly papules and plaques, located predominantly on the elbows, knees, and scalp (2, 3). Among patients with PSO, musculoskeletal inflammatory manifestations, involving peripheral joints, entheses, and axial skeleton, may be observed due to the occurrence of PSA (2). The latter may also be recognized in patients without a clinically evident PSO but with a suggestive family history; this is named PSA sine PSO (2).

Concerning the pathogenesis of psoriatic disease continuum, a complex and multidimensional model has been recently proposed linking the inflammatory mechanisms underlying both skin and joint manifestations (1–3). On a predisposing genetic background, a combination of environmental triggering factors (smoking, biomechanical stress, infections, or obesity), and an aberrant immune response may induce the development of a chronic inflammatory process (3). The resulting dysregulation of the innate and adaptive arms of the immune system may be also stimulated by cutaneous tissue, microbiome, and/or the entheses (3). Given the inflammatory pathogenesis of PSO and PSA, the assessment of cytokines may have the potential to accurately reflect the ongoing cellular processes and signaling pathways (4). Cytokines comprises interleukins (ILs), chemokines, interferons, and tumor necrosis factors. All these mediators have many pleiotropic effects which are strongly associated with the pathogenesis of PSA and PSO (5). Thus, a specific cytokine profile could better identify specific subtypes of patients in the context of psoriatic disease and, in particular, referring to a potential cytokine phenotype at increased risk of disease transition from PSO to PSA.

In this study, we aimed at evaluating the cytokine profile in patients with PSO, PsA with PSO, and PsA sine PSO, to identify possible differences or similarity in these groups of the same disease spectrum. We also assessed these biomolecules considering the presence of psoriatic nail involvement, the extension of skin disease, and the presence of obesity evaluating all involved patients

## Patients and methods

Consecutive patients were assessed among those attending the Dermatologic and/or Rheumatologic Clinics of the University of L’Aquila, L’Aquila, Italy and fulfilling classification criteria for PSO and/or PSA (6, 7). The treatment with systemic immunomodulating therapies for PSO and/or PSA was considered as a criterion of exclusion. The study was built on the assessment and comparison of three patient categories: i. PSO group, patients with evident skin involvement but without any musculoskeletal manifestation; ii. PSA with PSO group, patients with evident skin involvement and inflammatory musculoskeletal manifestations; iii. PSA sine PSO group, patients without an evident skin involvement but with inflammatory musculoskeletal manifestations. The cytokine profile of these patients was compared according to these three groups. In addition, patients were stratified considering the presence of psoriatic nail involvement, the extension of skin disease, and the presence of obesity. Severity and extension of skin involvement was evaluated by Psoriasis Area and Severity Index (PASI) and the nail involvement by Nail Area and Severity Index (NAPSI) (8, 9). Furthermore, obese patients were defined as those with body mass index (BMI) ≥ 30.

Written informed consents for all involved patients were collected allowing the use of retrieved findings for scientific purposes. The study protocol was submitted to the Local Ethics Committee (*Comitato Etico Azienda Sanitaria Locale 1 Avezzano/Sulmona/L’Aquila*, L’Aquila, Italy; protocol number 0204194/22) and the Internal Review Board of the University of L’Aquila (protocol number Internal Review Board University of L’Aquila 01/2022).

Following the current procedures for clinical samples, collected peripheral blood samples from patients were processed and analyzed. Collection tubes were rotated at 20°C for 30’ and centrifuged at 2000g for 15’, to spin down blood cells in order to separate the serum from cellular components. Patient sera were stored at −80°C into Eppendorf tubes to the execution of the analysis. The assessment was performed at a laboratory service provider equipped with the technology of Luminex (19 Plex) for biomarkers assay service (Labospace srl - Milano IT). The sera biomolecules were assessed by multiplex technology following the instructions of the manufacturer (Inflammation 20-Plex Human ProcartaPlex™ Panel, EPX200-12185-901). Twenty biomolecules were assessed with the inclusion of inflammatory cytokines (GM-CSF, IFN-γ, IL-1α, IL-1β, IL-6, IL-8, IL-12p70, IL-17A, IL-23, TNF, IFN-α, IL-4, IL-10, IL-13), inflammatory chemokines (IP-10, MCP-1, MIP-1α, MIP-1β), cell adhesion and cellular mediators (ICAM-1, E-selectin, P-Selectin). The interpretation of results was performed by using a dedicated software.

Firstly, a descriptive statistic was made of patient clinical manifestations. Given their distribution, continuous variables were presented as mean/standard deviation (SD) or median/interquartile range (IQR), as appropriate. One-Way ANOVA test was used to compare the groups, considering the main categories and the selected clinical features (psoriatic nail involvement, PASI ≥ 10, BMI ≥ 30). After that, possible correlations were estimated among biomolecules, NAPSI, PASI, and BMI by Spearman’s rank correlation coefficient. Two-sided P values < 0.05 were considered as being statistically significant. Prism – GraphPad version 8.0 was used for all analyses.

## Results

In this evaluation, 80 consecutive naïve patients were assessed, before the administration of systemic immunomodulating therapies, as reported in **Table 1**. In the PSO group, 19 patients were comprised (age: 48.6 ± 15.8 years, male sex 47.4%, BMI: 26.8 ± 5.2). Psoriatic onychopathy was observed in 52.6% of the assessed patients with PSO, NAPSI resulted to be median 10.5 (50.0) and PASI was median 5.9 (18.3). No tender or swollen joints were recorded in these patients. Forty-one PSA with PSO patients were assessed (age: 54.7 ± 11.1, male sex: 29.3%, BMI: 27.4 ± 6.0). In this group, 70.7% of patients showed psoriatic onychopathy, NAPSI resulted to be 19.0 (68.0) and PASI 2.5 (46.0). These patients affected by PSA with PSO were also characterized by tender [8.0 (46.0)] and swollen joints [1.0 (5.0)]. In addition, 20 patients with PSA sine PSO were evaluated (age: 56.6 ± 12.9, male sex: 15.0%, BMI: 26.2 ± 3.5). These patients did not show skin involvement but tender [8.0 (25.0)] and swollen joints [1.0 (4.0)].

**Table 1:** Descriptive statistics of assessed patients with PSO, PSA with PSO, and PSA sine PSO.

	PSO	PSA with PSO	PSA sine PSO
	19	41	20
Age, years, mean ± SD	48.6 ± 15.8	54.7 ± 11.1	56.6 ± 12.9
Male Sex, %	47.4%	29.3%	15.0%
BMI, mean ± SD	26.8 ± 5.2	27.4 ± 6.1	26.2 ± 3.5
BMI≥ 30	36.8%	34.1%	35.0%
Dermatologic assessment			
Plaque type PSO, %	89.5%	78.1%	0.0%
Inverse genital PSO, %	26.4%	17.1%	0.0%
Palmo plantar PSO, %	26.3%	24.4%	0.0%
Guttate PSO, %	36.8%	34.1%	0.0%
Psoriatic onychopathy, %	52.6%	70.7%	0.0%
NAPSI, median (range)	10.5 (50.0)	19.0 (68.0)	0.0 (0.0)
PASI, median (range)	5.9 (18.3)	2.5 (46.0)	0.0 (0.0)
Rheumatologic assessment			
Tender joints, median (range)	0.0 (0.0)	8.0 (46.0)	8.0 (25.0)
Swollen joints, median (range)	0.0 (0.0)	1.0 (5.0)	1.0 (4.0)
VAS pain, median (range)	0.0 (5.0)	5.5 (10.0)	5.0 (8.0)
LEI, median (range)	0.0 (0.0)	2 (4.0)	2 (3.0)

PSO, psoriasis; PSA, psoriatic arthritis; BMI, body mass index; PASI, Psoriasis Area and Severity Index; NAPSI, Nail Area and Severity Index; VAS, visual analogue scale; LEI, Leed Enthesitis Index.

**Figure 1:**
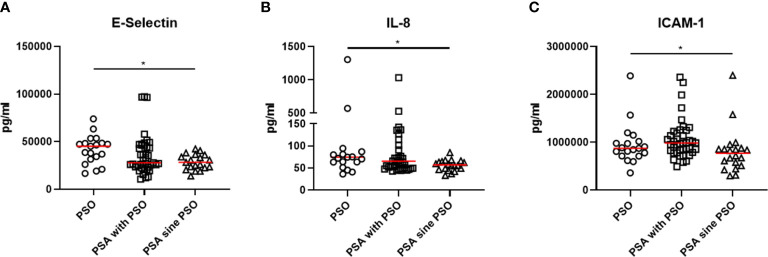
**(A, B)** increased levels of E-selectin and IL-8 were retrieved in patients with PSO than patients with PSA with PSO or patients with PSA sine PSO. **(C)** significant higher levels of ICAM-1 characterized patients with PSA than patients with PSO and patients with PSA sine PSO. *: p < 0.05. IL, interleukin, PSO, psoriasis; PSA, psoriatic arthritis; ICAM-1, Intercellular Adhesion Molecule 1.

**Figure 2:**
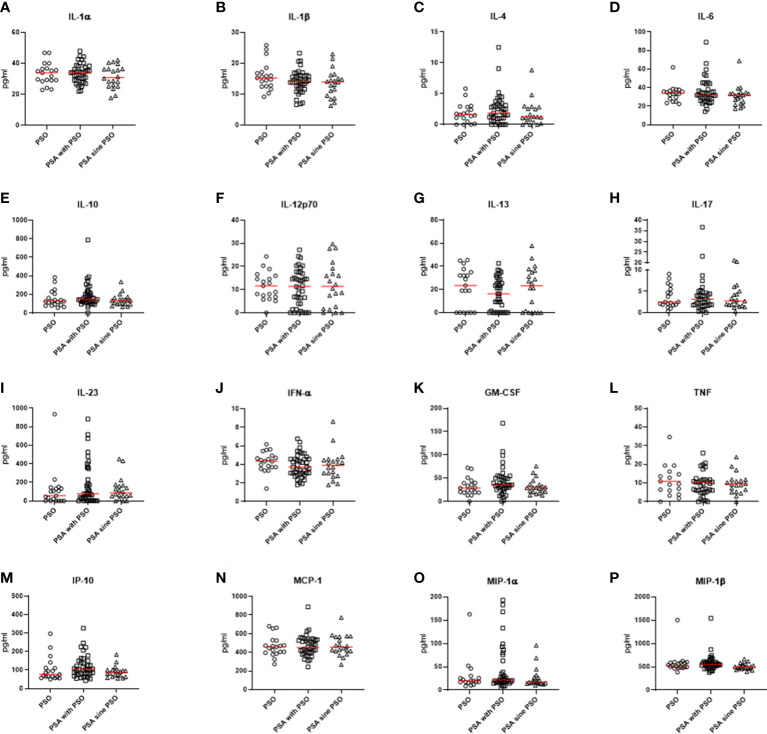
**(A-P)** No significant differences were observed comparing pro-inflammatory and anti-inflammatory cytokines, inflammatory chemokines, and cell adhesion and cellular mediators in patients with PSO, PSA with PSO, and PSA sine PSO. PSO, psoriasis; PSA, psoriatic arthritis; IL, interleukin; IFN-α, interferon α; GM-CSF, granulocyte-macrophage colony-stimulating factor; TNF, tumor necrosis factor; IP-10, interferon gamma-induced protein 10; MCP-1, Monocyte Chemoattractant Protein-1; MIP, Macrophage Inflammatory Proteins.

Comparing the cytokine profile, some significant differences were observed in the three groups, as shown in **Figure 1**. Patients with PSO were characterized by a significant increase of levels of E-selectin (p=0.021) than patients with PSA with PSO or patients with PSA sine PSO. Similarly, levels of IL-8 (0.041) were significantly increased in patients with PSO in respect to others. In the PSA with PSO group, significant enhanced levels of ICAM-1 were detected (p=0.009) than patients with PSO and patients with PSA sine PSO. No additional significant differences were retrieved comparing other inflammatory cytokines, inflammatory chemokines, and cell adhesion and cellular mediators in patients with PSO, PSA with PSO, and PSA sine PSO. These results are summarized in **Figure 2**. The assessment of P-selectin and IFN-γ did not produce any result, these biomolecules appeared to be not expressed.

**Figure 3:**
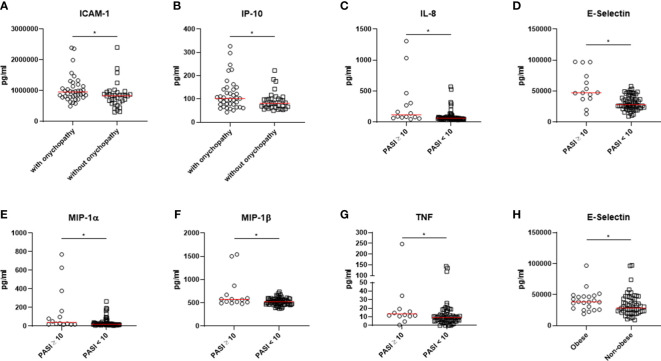
**(A, B)** Patients characterized by psoriatic onychopathy showed significant higher levels of ICAM-1 and IP-10. **(C-G)** Higher values of IL-8, TNF, E-selectin, MIP-1α, and MIP-1β were observed in patients with PASI ≥ 10. **(H)** Patients with BMI ≥ 30 were characterized by significantly higher levels of E-selectin. *: p < 0.05. PSO, psoriasis; PSA, psoriatic arthritis; ICAM-1, Intercellular Adhesion Molecule 1; IP-10, interferon gamma-induced protein 10; IL, interleukin; MIP, Macrophage Inflammatory Proteins; PASI, Psoriasis Area and Severity Index; BMI, body mass index.

After that, our results were stratified according to selected clinical characteristics (i.e., psoriatic onychopathy, PASI ≥ 10, BMI ≥ 30) considering all involved patients, as shown in **Figure 3**. Patients characterized by psoriatic onychopathy showed significant enhanced values of ICAM-1 (p=0.010) and IP-10 (p=0.030) than others. Assessing possible associations among these results, a positive correlation between IL-8 and NAPSI was observed (R=0.233, p=0.046). Similarly, NAPSI directly correlated with ICAM-1 (R=0.235, p=0.045), and with MIP-1α (R=0.319, p=0.006). Furthermore, the results were analyzed based on the extension of skin involvement. In patients with PASI ≥ 10, significantly increased values of IL-8 (p=0.004) and TNF (p=0.013) were observed than others. Additionally, these patients were distinguished by significantly enhanced values of E-selectin (p=0.004), MIP-1α (p=0.003), and MIP-1β (p=0.039). Possible correlations between values of assessed biomolecules and PASI were also exploited. Direct correlations were also observed analyzing PASI and IL-8 (R=0.360, p=0.002) and PASI and TNF (R=0.238, p=0.042). Moreover, PASI positively correlated with E-selectin (R=0.649, p<0.001), MIP-1α (R=0.355, p=0.002), and MIP-1β (R=0.244, p=0.037), respectively. Finally, our results were assessed considering the presence of obesity. In patients with BMI ≥ 30, significantly higher levels of E-selectin were pointed out (p=0.035) than others. In addition, a monotonic effect between BMI and E-selectin was reported (R=0.339, p=0.003).

## Discussion

Comparing naïve patients with PSO, PSA with PSO, and PSA sine PSO, our data suggested a similar cytokine profile according to a psoriatic disease continuum. No differences were retrieved about IL-23/IL-17-pathway in these patients. The prominent role of this inflammatory axis has been proposed in this context of psoriatic disease (10). More specifically, IL-23 is a cytokine member of IL-12 superfamily, showing both immunoregulatory activity and effector functions (11). IL-23 may induce the production of IL-17 and TNF which contribute to both synovial inflammation and skin lesions (3). The clinical relevance of the inhibition of the IL-23/IL-17-axis and TNF has been clearly pointed out in patients with PSO and PSA (3, 11, 12). Furthermore, no additional significant differences were retrieved in the assessment of further inflammatory biomolecules, including IL-1β, IL-6, IL-10, and IL-13. Taking together these results, the cytokine profile of the group of PSA sine PSO did not significantly differ than those with evident PSO, suggesting these patients as an extreme of the psoriatic disease continuum. In addition, these results may provide a further rationale behind the clinical findings concerning the similar subclinical abnormalities of nails and musculoskeletal features observed in these patients, which may reinforce the concept of the psoriatic disease continuum (13–15). In addition, although further confirmatory studies are needed, these results may highlight possible mechanistic biomarkers which could better identify non-responder patients to the treatment since these biomolecules may more closely reflect the manipulated pathogenic pathways (4, 5)

Assessing our data, we found some differences in biomolecules in these groups of patients. Higher levels of IL-8 characterized patients with PSO than those with PSA. IL-8 is also known as “neutrophil chemotactic factor*”* and has two main functions, primarily inducing the chemotaxis of neutrophils and stimulating their phagocytotic activity (16). It has been suggested as responsible for the exacerbation of PSO, enhancing expression of IL-17 in keratinocytes and favoring the development of skin lesions (17). In fact, IL-8 correlated with the extension of skin involvement in our cohort of patients. In addition, in our study, levels of E-selectin were higher in PSO group in respect to PSA. This molecule, also identified as CD62 antigen-like family member E (CD62E), is a selectin cell adhesion mediator which is found on activated endothelial cells activated (18). In this context, previous evidence showed high levels of E-selectin in patients with PSO relating to the extension of skin involvement (18, 19). The presence of E-selectin on the surface of endothelial cells may lead to the interaction of between these cells and circulating leukocytes. Thus, a trans-endothelial migration of leukocytes from the blood to the epidermis may be induced, contributing to the inflammation of the psoriatic lesions (18–20). Furthermore, we showed higher values of ICAM-1 in PSA with PSO group than others. This molecule is codified as a cell surface glycoprotein. It is generally found on both endothelial cells and immune cells and contributes to the migration of leukocytes toward the tissue inflammatory infiltrate. The overexpression of ICAM-1 has been previously documented in synovial fluids collected from patients with PSA (21). In addition, the inhibition of the interaction between LFA-1 and ICAM-1 may induce the occurrence of arthritis in patients with PSO, possibly altering the balance of leucocyte extravasation (22). Finally, considering that IL-8, E-selectin, and ICAM-1 are all involved in angiogenesis, it is also possible to speculate that the hyperactivation of angiogenetic pathways could be implicated in patients with PSO in respect to those sine PSO. In fact, the crucial role of angiogenesis in PSO has been already suggested (23), but its role in PSA sine PSO should be better evaluated.

After these evaluations among main patient groups, we stratified our results according to relevant clinical features in the context of psoriatic disease (i.e., presence of psoriatic nail involvement, PASI ≥ 10, BMI ≥ 30) considering the whole cohort. Patients characterized by psoriatic onychopathy showed significant higher levels of ICAM-1 and IP-10. The latter, also named CXCL10, has been related to the occurrence of development of joint manifestations in patients with PSO, which are associated with psoriatic nail involvement (24–26). However, additional data are needed to fully elucidate the pathogenic involvement IP-10 in the development of psoriatic onychopathy and possibly arthritis. Moreover, the severity of psoriatic onychopathy correlated with IL-8, ICAM-1, and with MIP-1α, suggesting a possible more specific involvement of these mediators in the occurrence of such clinical manifestation. In addition, in patients with PASI ≥ 10, we observed significantly higher values of IL-8, TNF, E-selectin, MIP-1α, and MIP-1β than others. In previous studies, the expression of these biomolecules was associated with the severity of skin involvement in PSO (27, 28). Moreover, in patients with BMI ≥ 30, significantly higher levels of E-selectin were pointed out than others. This finding may parallel the available literature since the endothelial dysfunction due to expression of E-selectin is to be considered a consequence of the metabolic alterations of obesity (29, 30).

Overall, our results may point out some pathogenic differences according to relevant clinical features, psoriatic nail involvement, extensive skin involvement, and obesity, in the transition from PSO to PSA (15). Nevertheless, the available evidence suggests that additional clinical variables may be associated with transition from skin involvement to synovio-entheseal disease in psoriatic disease continuum, such as first-degree relative with PSA, joint biomechanical stress, and microbiome-related event (15). However, additional studies are warranted to clarify further clinical differences in this context. The relevance of sex-related differences in patients with PSA has been recently highlighted (31), but their influence in inducing the transition from PSO to PSA should be fully investigated. Also, the possible impact of racial differences should be taken into account to design more studies in regard to this topic considering that some differences were shown according to such features (32, 33). In addition, given that both intestinal microbiome perturbations and obesity are linked with development of PSA (15, 34), the role of diet and associated weight loss may be carefully evaluated in patients with PSO who are considered at high risk to develop PSA (34). Finally, specific designed prospective studies are also advocated to entirely evaluate the potential impact of systemic therapies, which are administered for the skin involvement, in attenuating the occurrence of PSA in patients with PSO (34). In fact, the use of biologic agents in patients with plaque PSO appeared to delay or reduce the risk of incident PsA (35).

Some limitations may be recognized in our study; our findings would be cautiously interpretated. Despite the assessment of naïve patients, the somewhat small number of assessed patients and the single center design may impair the generalization of the results. In fact, our study may be underpowered to detect some differences between PSO and PSA, considering the heterogeneous clinical courses and possible fluctuation of disease severity over time. In addition, the serum cytokine profile could not fully mirror the histopathology of the affected tissues by psoriatic disease, suggesting the need of specifically designed evaluations on both skin as well as synovium of these patients. Therefore, the hypothesis-generating nature of our study should be recognized and future confirmatory works are warranted to fully elucidate these issues and their clinical consequences.

In conclusion, a similar cytokine profile was observed in naïve patients with PSO, PSA with PSO, and PSA sine PSO, reinforcing the idea of psoriatic disease according to a pathogenic continuum. Our data also suggested some differences which may underly possible pathogenic diversities leading to the clinical heterogeneity of these patients. Further data are needed to entirely clarify these issues and their clinical consequences in improving the management of such patients of the psoriatic disease continuum.

## Data availability statement

The original contributions presented in the study are included in the article/supplementary material. Further inquiries can be directed to the corresponding author.

## Ethics statement

The studies involving human participants were reviewed and approved by Comitato Etico Azienda Sanitaria Locale 1 Avezzano/Sulmona/L’Aquila, L’Aquila, Italy. The patients/participants provided their written informed consent to participate in this study.

## Author contributions

All authors made substantial contributions to the conception or design of the work, the acquisition and interpretation of data. All authors contributed to the critical review and revision of the manuscript and approved the final version. All the authors agreed to be accountable for all aspects of the work.
